# Supplementary material for: DiffVar: a new method for detecting differential variability with application to methylation in cancer and aging
Source: Genome Biol. 2014 Sep 23;15(9):465. doi: 10.1186/s13059-014-0465-4 (PMC4210618; doi:10.1186/s13059-014-0465-4)
Supplement: Additional file 1: — Statistical model details of DiffVar. [file 13059_2014_465_MOESM1_ESM.pdf]

DiffVar: a new method for detecting differential variability with application to methylation in cancer and aging Supplementary Text

Belinda Phipson Alicia Oshlack

1 Calculating absolute or squared residuals

Let y_{ijk} denote the M-value for the ith sample, $i = 1, \ldots, n_k$, the jth CpG site, $j = 1, \ldots, 482\ 421$ and the kth group, $k = 1, \ldots, K$. Here n_k is the sample size for the kth group and K is the total number of groups. For a two group comparison, e.g. cancer vs normal, $K = 2$. Note that each CpG site is analysed independently of the other CpGs.

The first step in the method is to calculate absolute or squared residuals for each observation in each of the K groups for each CpG. For the jth CpG site, the mean M-value for the kth group is calculated by

$$\bar{y}_{jk} = \frac{\sum_{i=1}^{n_k} y_{ijk}}{n_k}.$$

Absolute deviations, or residuals, are calculated as

$$z_{ijk} = |y_{ijk} - \bar{y}_{jk}| \times \sqrt{\frac{n_k}{n_k - 1}},$$

where $\sqrt{n_k/(n_k - 1)}$ is a leverage factor which takes into account unequal sample sizes. This ensures that groups with larger samples sizes are not biased towards detecting larger variances compared to groups with smaller sample sizes. If squared deviations are required,

$$z_{ijk} = (y_{ijk} - \bar{y}_{jk})^2 \times \sqrt{\frac{n_k}{n_k - 1}}.$$

2 Calculating moderated t statistics

The z_{ijk}'s capture how much each sample deviates from the group mean. Hence groups that are more variable will have larger z_{ijk}'s on average, and groups that are more consistent will have smaller z_{ijk}'s on average. Let the true mean of the z_{ijk} for group k and CpG site j be denoted $\mu_{z_{jk}}$. Thus, for two groups, testing the null hypothesis $H_0 : \mu_{z_{j1}} = \mu_{z_{j2}}$ effectively tests whether the two group variances are equal, or $H_0 : \sigma^2_{j1} = \sigma^2_{j2}$. Here σ^2_{jk} is the unknown true variance of the y_{ijk}'s for group k and CpG site j. For the jth CpG site and the kth group, the mean absolute (or squared) deviation, $\bar{z}_{jk}$, is given by

$$\bar{z}_{jk} = \frac{\sum_{i=1}^{n_k} z_{ijk}}{n_k}.$$

In general, let $\mathbf{z}_j^T = (z_{1j}, \ldots, z_{nj})$ be the vector of absolute or squared deviations for CpG site j, where $n = n_1 + \ldots + n_K$ is the total number of samples in the experiment. We can fit a linear model,

$$E(\mathbf{z}_j) = X\boldsymbol{\beta}_j, \qquad (1)$$

where X is a design matrix of full column rank and $\boldsymbol{\beta}_j$ is a vector of coefficients. For the two group case, with no additional covariates, $\boldsymbol{\beta}_j^T = (\beta_{j0}, \beta_{j1})$, where β_{j0} is the intercept and β_{j1} is the regression coefficient. Here $\hat{\beta}_{j1}$ is estimated as the difference between the mean absolute or squared deviation between group 1 and 2,

$$\hat{\beta}_{j1} = \bar{z}_{j1} - \bar{z}_{j2}.$$

More generally, in matrix terminology, a vector of regression coefficients can be estimated by

$$\hat{\boldsymbol{\beta}}_j = (X^T X)^{-1} X^T \mathbf{z}_j.$$

The variance of the absolute or squared deviations for the jth CpG site is denoted $s_{z_j}^2$ and are the residuals obtained from fitting the linear model in Eqn. 1.

The classic Levene's test (Levene, 1960) uses squared deviations and calculates ordinary t-statistics in the case of a two group comparison, or an ANOVA in the case of $K > 2$. For a 2 group comparison, the ordinary t test statistic for CpG site j is

$$t_j = \frac{\hat{\beta}_{j1}}{s_{zj}\sqrt{\nu}}.$$

where ν is the appropriate diagonal element from the positive definite matrix $(X^T X)^{-1}$. Two-sided p-values can be computed from the t distribution with degrees of freedom equal to $d_j = n - p$, where p is the number of parameters estimated in the linear model.

It is well established in the genomics field that performing an ordinary t-test results in many false positives, particularly for studies with smaller sample sizes (Efron *et al.*, 2001; Tusher, Tibshirani and Chu, 2001; Lönnstedt and Speed, 2002; Broberg, 2003; Wright and Simon, 2003). Hence, rather than calculating ordinary t-tests, once the absolute or squared deviations have been obtained, moderated t-statistics (Smyth, 2004), which employ empirical Bayes shrinkage of the $s_{z_j}^2$, are calculated rather than ordinary t-statistics. For full hierarchical model details and derivation of the moderated t-statistic please refer to Smyth (2004). The moderated t-statistic is defined as

$$\tilde{t}_j = \frac{\hat{\beta}_{j1}}{\tilde{s}_{zj}\sqrt{\nu}},$$

where

$$\tilde{s}_{zj}^2 = \frac{d_0 s_{z0}^2 + d_j s_{z_j}^2}{d_0 + d_j}$$

are the squeezed variances. The d_0 and s_{z0}^2 are hyperparameters of the hierarchical model that can be estimated using empirical Bayes estimation procedures, see Smyth (2004) for more details. For differentially variable CpG sites, $\tilde{t}_j$ follows a scaled t distribution with degrees of freedom $d_0 + d_j$. For CpG sites with no differences in variances, $\tilde{t}_j$ follows an unscaled t distribution with degrees of freedom $d_0 + d_j$.

Once p-values are obtained from the moderated t statistics, they are adjusted for multiple testing using the method of Benjamini and Hochberg (1995).

3 Thresholding on the log ratio of group variances

In addition to a p-value cut-off, a cut-off can be specified on the log ratio of the estimated group variances, defined as

$$\text{LogVarRatio} = \log\left(\frac{s_{j1}^2}{s_{j2}^2}\right).$$

Specifying a LogVarRatio of at least $|\log(2)|$ means that the variance of one group is at least twice that of the second group. In our cancer datasets, we specified a LogVarRatio cut-off of at least $|\log(5)|$. The LogVarRatio's are symmetric about zero, with negative values meaning that the second group is more variable than the first, and positive values mean that the first group is more variable than the second.

References

[1] Benjamini, Y., and Hochberg, Y. (1995). Controlling the false discovery rate: a practical and powerful approach to multiple testing. *Journal of the Royal Statistical Society Series B*, **57**: 289–300.

[2] Broberg, P. (2003). Statistical methods for ranking differentially expressed genes. *Genome Biology*, **4**: R41.

[3] Efron, B., Tibshirani, R., Storey, J.D., and Tusher, V. (2001). Empirical Bayes analysis of a microarray experiment. *Journal of the American Statistical Association*, **96**: 1151–1160.

[4] Levene, H. (1960). Robust tests for equality of variances. In *Contributions to Probability and Statistics*. Stanford University Press, Stanford, 278–292.

[5] Lönnstedt I., and Speed, T.P. (2002). Replicated microarray data. *Statistica Sinica*, **32**: 461–552.

[6] Smyth, G.K. (2004). Linear models and empirical Bayes methods for assessing differential expression in microarray experiments. *Statistical Applications in Genetics and Molecular Biology*, **3**: Article 3.

[7] Tusher, V.G., Tibshirani, R., and Chu, G. (2001). Significance analysis of microarrays applied to ionizing radiation response. *Proceedings of the National Academy of Sciences of the United States of America*, **98**: 5116–5121.

[8] Wright, G.W., and Simon, R.M. (2003). A random variance model for detection of differential gene expression in small microarray experiments. *Bioinformatics*, **19**(18): 2448–2455.
