# Supplementary material for: DiffVar: a new method for detecting differential variability with application to methylation in cancer and aging
Source: Genome Biol. 2014 Sep 23;15(9):465. doi: 10.1186/s13059-014-0465-4 (PMC4210618; doi:10.1186/s13059-014-0465-4)
Supplement: Additional file 2: — Additional figures S1–S8. [file 13059_2014_465_MOESM2_ESM.pdf]

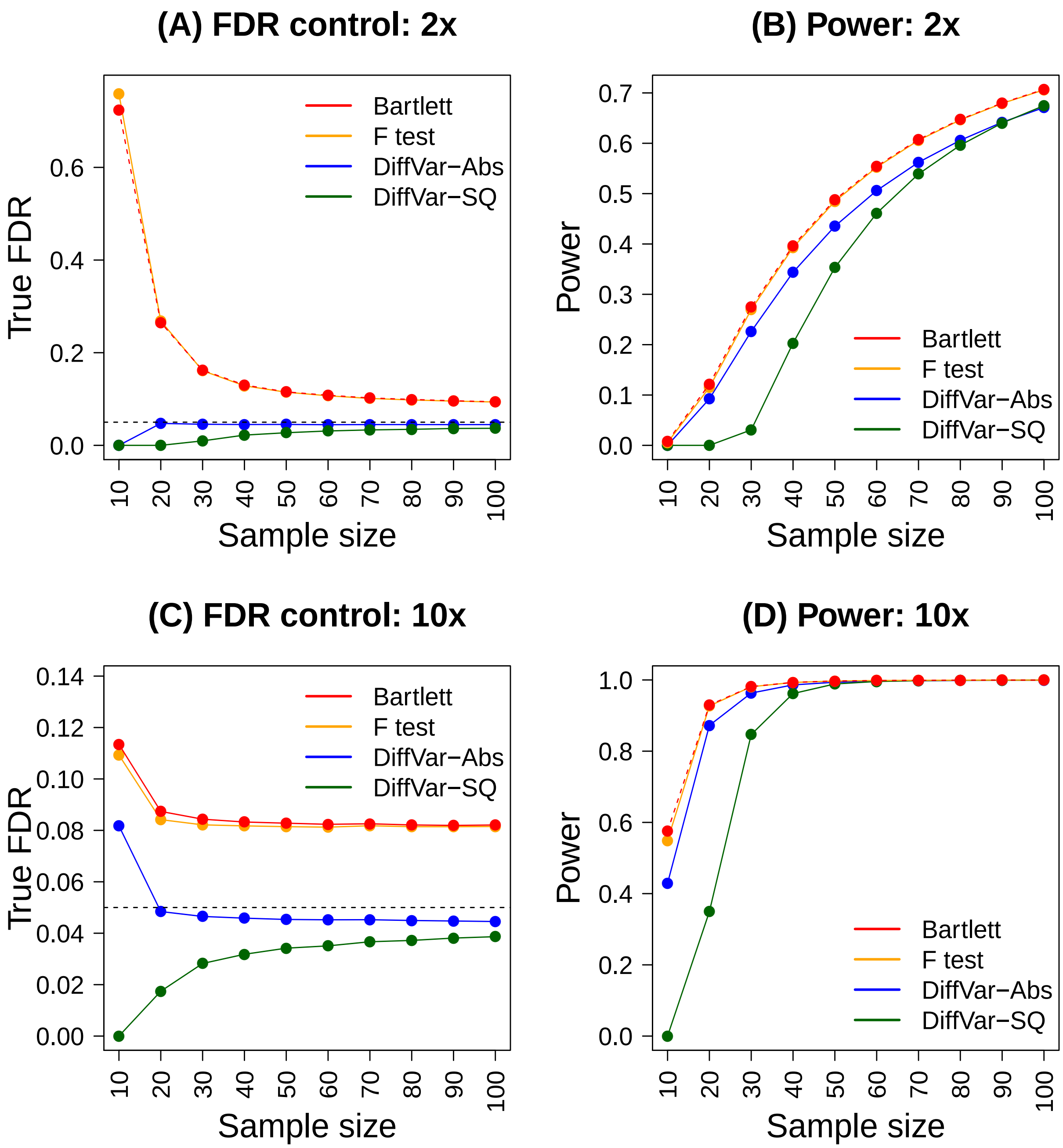


Figure S1: **Comparative results for simulations with differing levels of variability and differing sample sizes.** The top panel shows results for Group 2 twice as variable as Group 1 for 1000 features, and the bottom panels shows results for Group 2 ten times more variable than Group 1. Plots (A) and (C) show the control of the false discovery rate (FDR) for the four methods over ten different sample sizes. The Bartlett and F test fail to control the FDR at a 5% cut-off (the black dotted line). DiffVar (abs) has slightly elevated FDR when $n = 10$ and the variability is large, but achieves the required rate for all other sample sizes. Plots (B) and (D) show how powerful each method is to detect differentially variable features for different sample sizes. DiffVar (sq) has the least power with the F and Bartlett test showing the most power. DiffVar (abs) shows the best balance between FDR control and power.

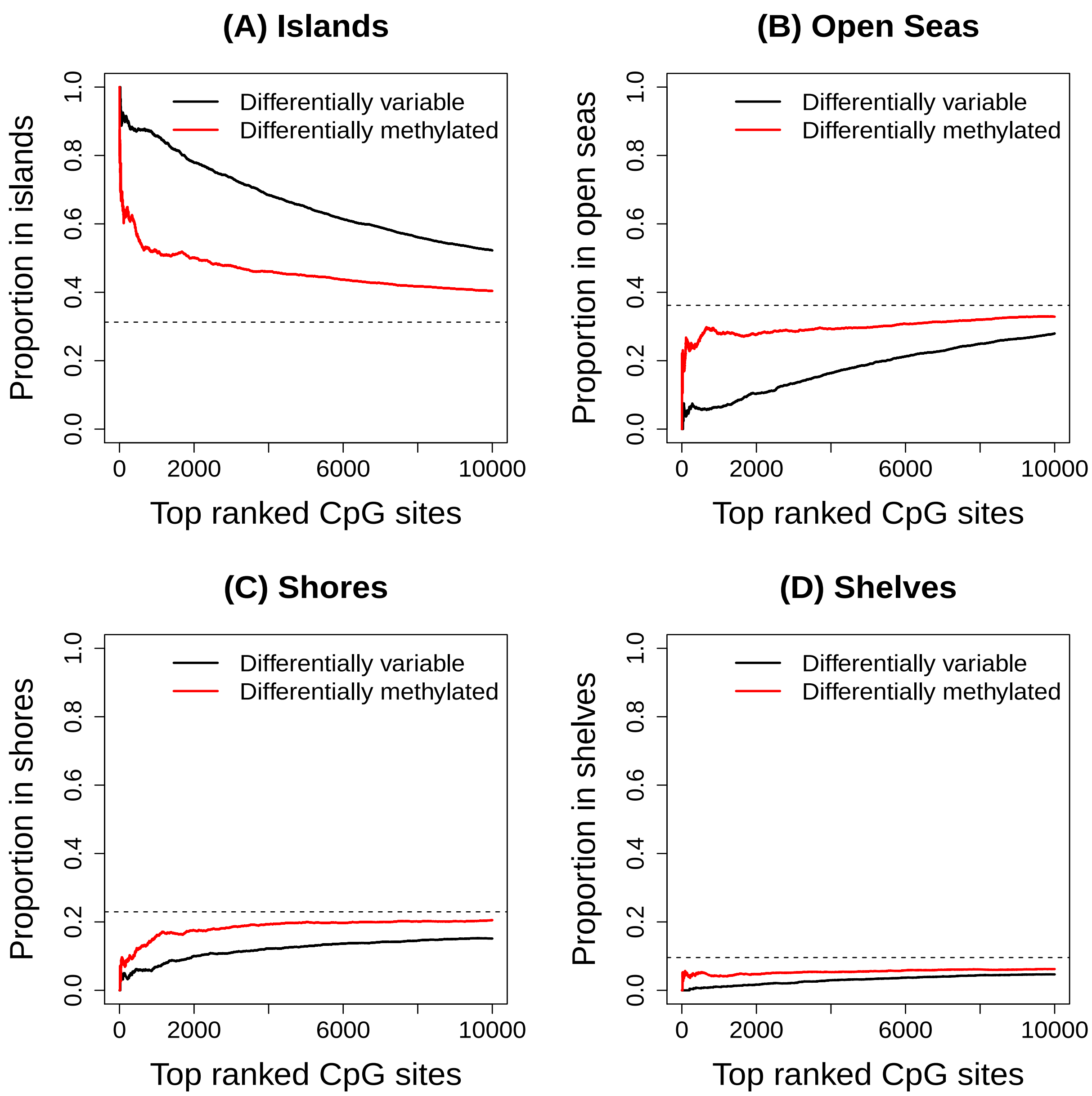


Figure S2: **Proportion of significant differentially variable CpG sites present in different genomic regions in the lung cancer dataset.** Plot (A) shows the proportion of top 10 000 ranked CpGs in islands, (B) in open seas, (C) in shores and (D) in shelves in the lung cancer dataset. The horizontal dashed line represents the overall proportion of probes interrogating the particular genomic region on the array.

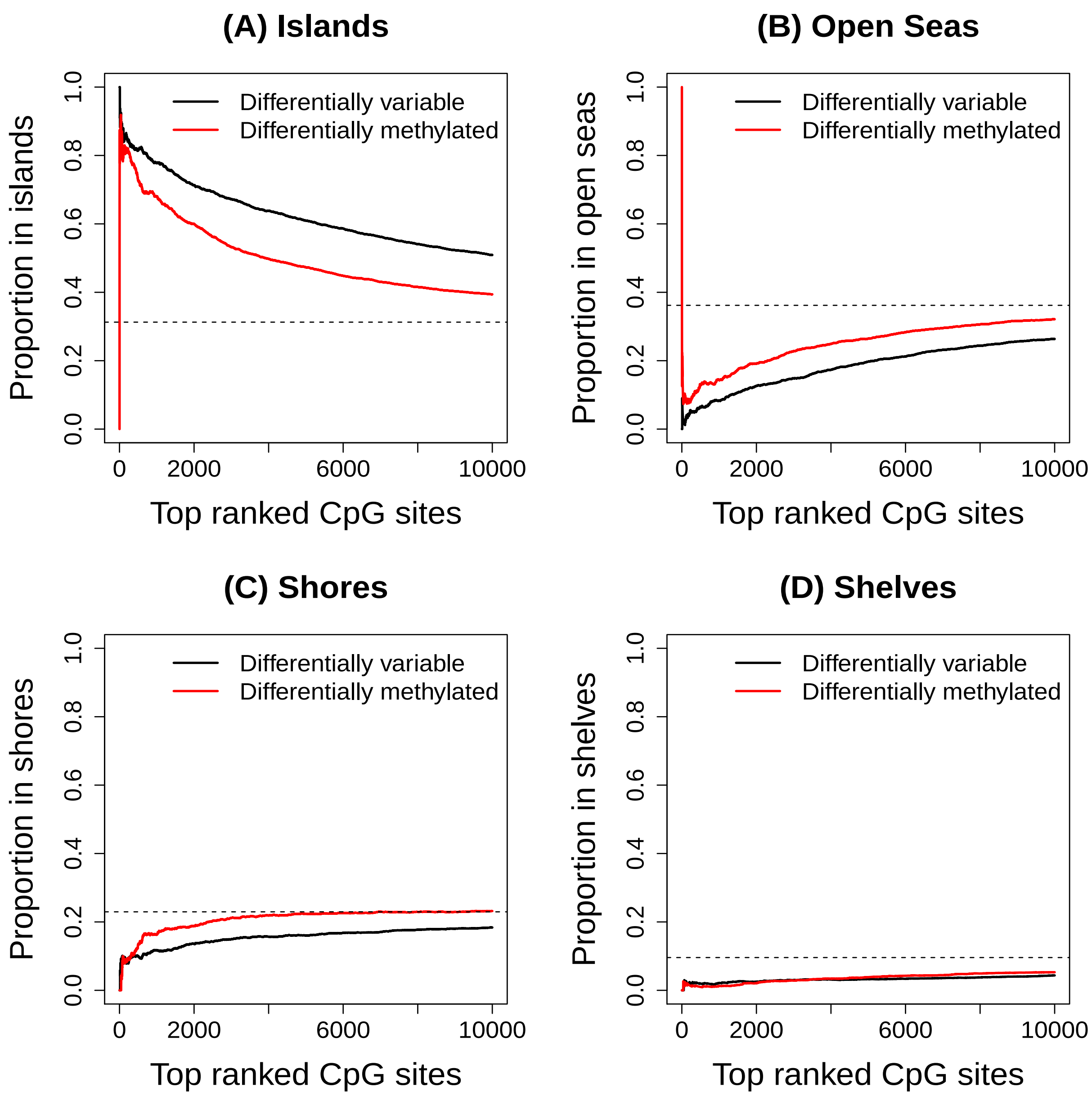


Figure S3: **Proportion of significant differentially variable CpG sites present in different genomic regions in the prostate cancer dataset.** Plot (A) shows the proportion of top 10 000 ranked CpGs in islands, (B) in open seas, (C) in shores and (D) in shelves in the prostate cancer dataset. The horizontal dashed line represents the overall proportion of probes interrogating the particular genomic region on the array.

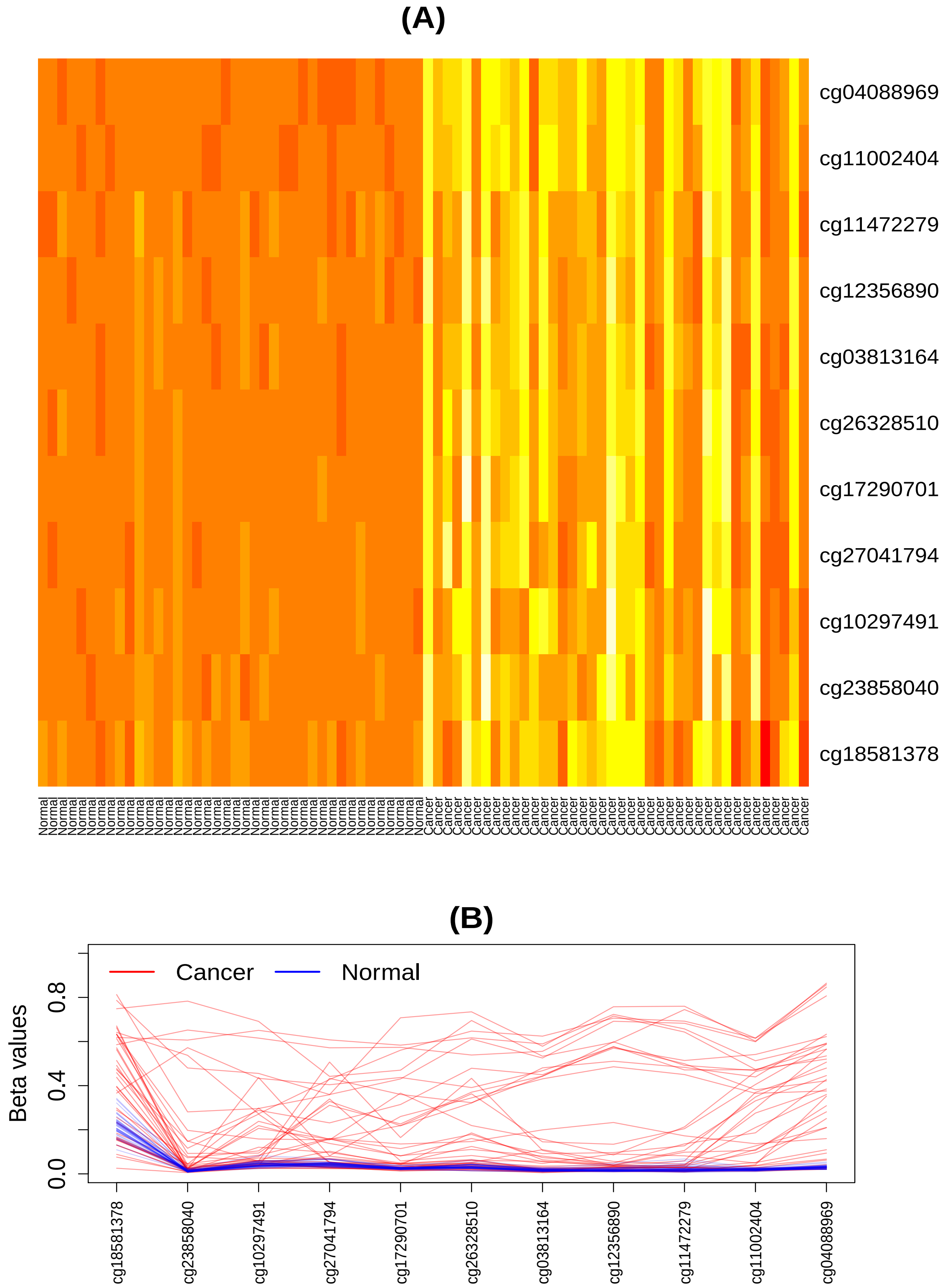


Figure S4: **Methylation levels of a CpG island in the kidney cancer dataset.** Eighty samples from the normal and cancer groups were randomly selected, with CpG sites ordered by genomic location. (A) Heatmap showing cancer groups are heterogenous between individuals in the cancer group, however within an individual they are highly correlated. (B) Each line represents the methylation status of an individual across ordered CpG sites in the CpG island.

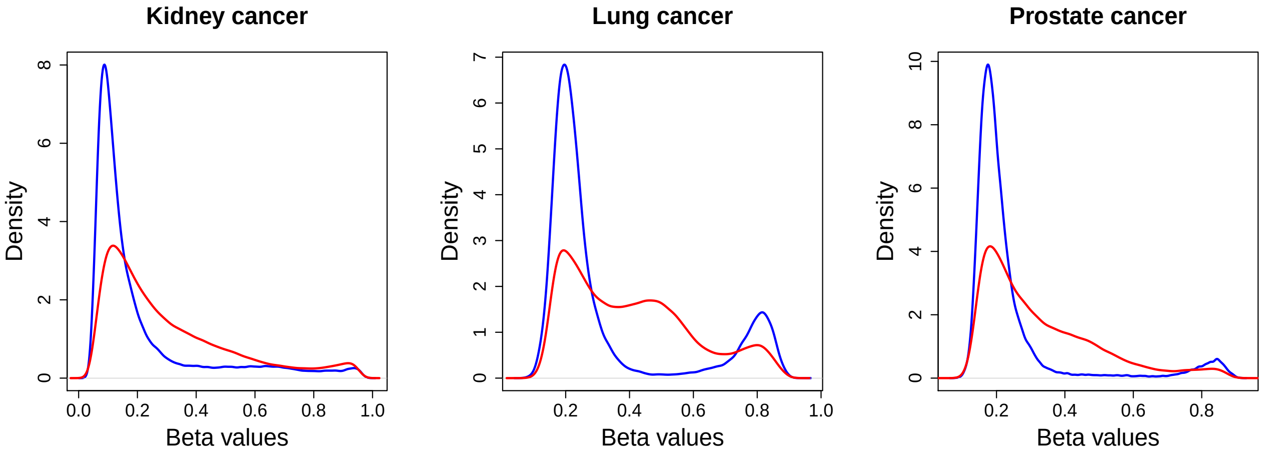


Figure S5: **Density plots of top 1000 DV CpG islands in the three cancer datasets.** The CpG islands tend to be un-methylated in normal samples (blue line). The methylation profile of the top CpG islands is moving towards methylated in the cancers (red line).

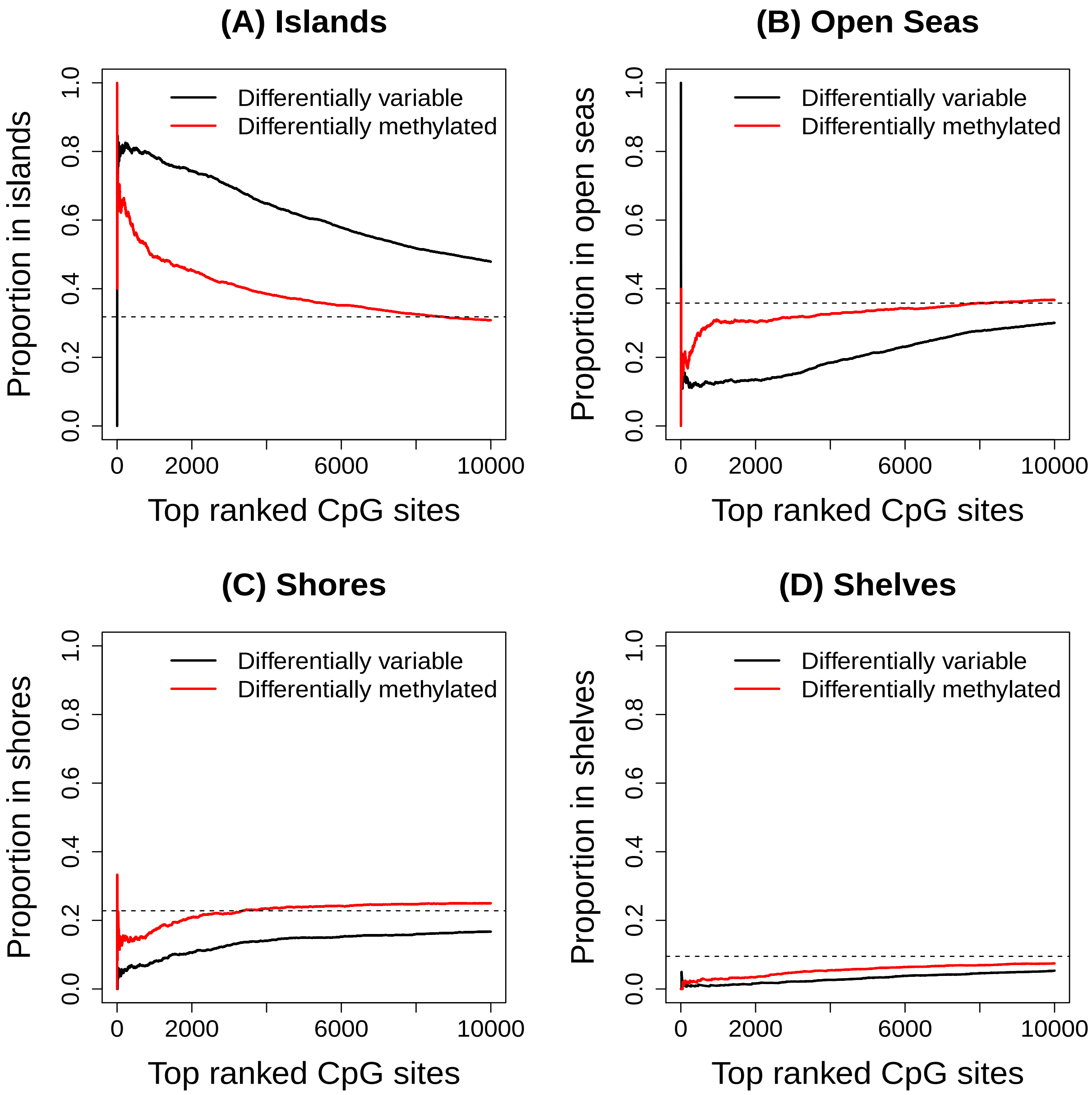


Figure S6: **Proportion of significant differentially variable CpG sites present in different genomic regions in the uterine cancer dataset.** Plot (A) shows the proportion of top 10 000 ranked CpGs in islands, (B) in open seas, (C) in shores and (D) in shelves in the uterine cancer dataset. The horizontal dashed line represents the overall proportion of probes interrogating the particular genomic region on the array.

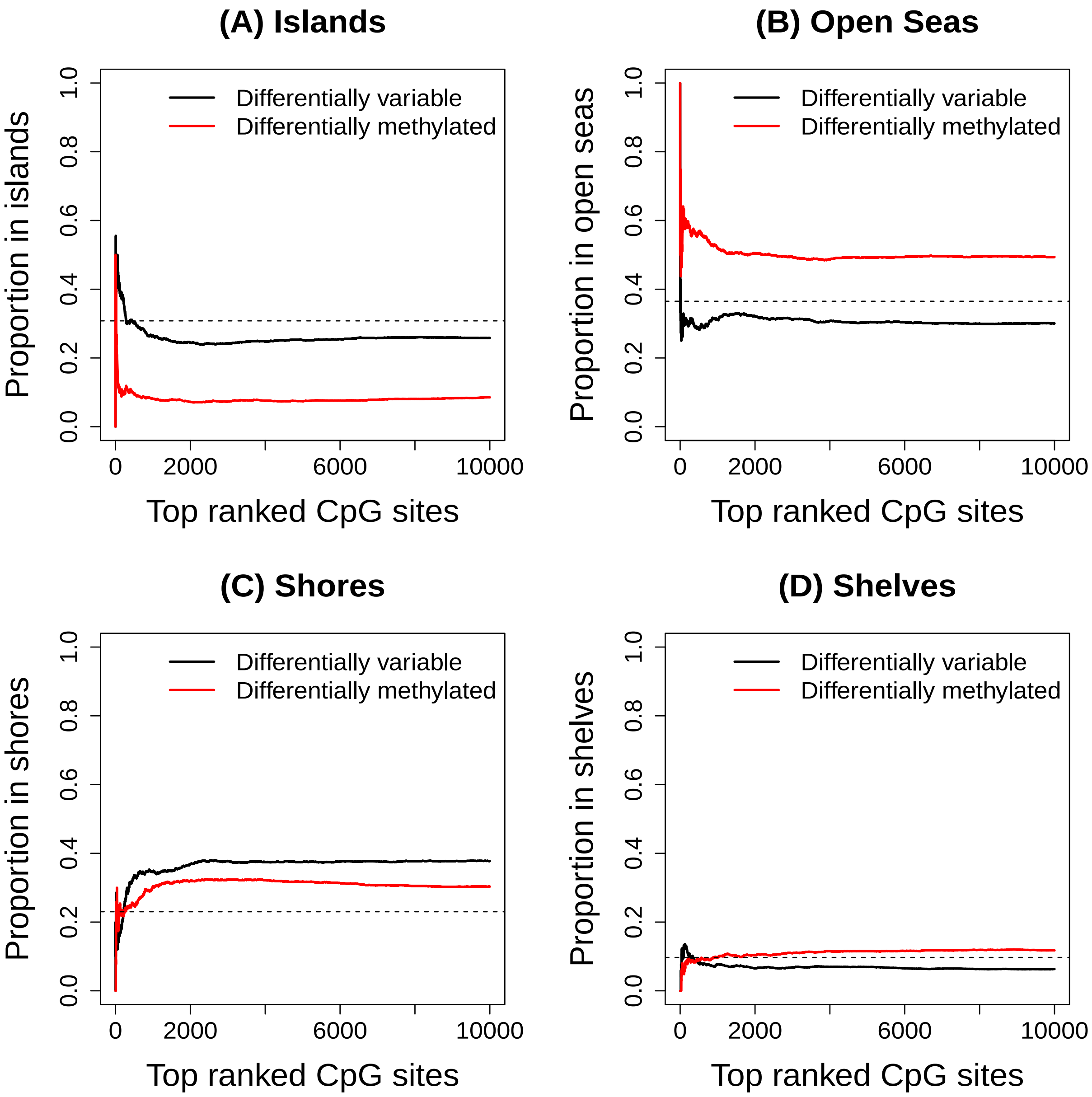


Figure S7: **Proportion of significant differentially variable CpG sites present in different genomic regions in the aging dataset.** Plot (A) shows the proportion of top 10 000 ranked CpGs in islands, (B) in open seas, (C) in shores and (D) in shelves in the aging dataset. The horizontal dashed line represents the overall proportion of probes interrogating the particular genomic region on the array.

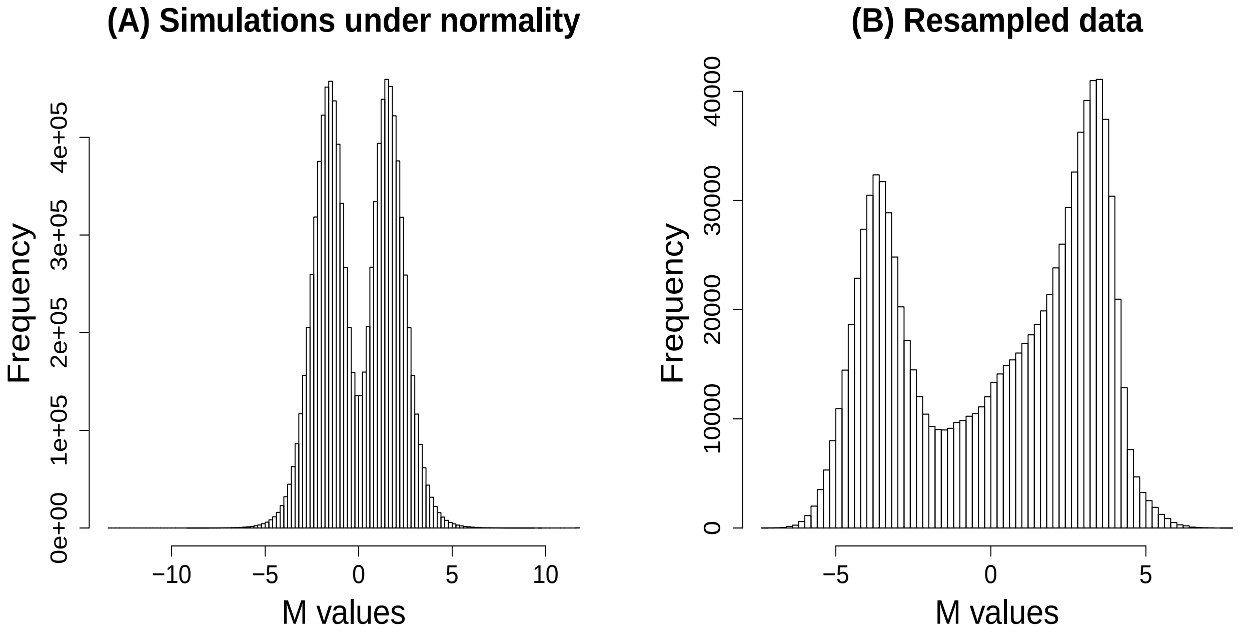


Figure S8: **Histogram of M values under the two simulation strategies.** (A) Half the CpG sites are methylated and half are unmethylated, resulting in two equal peaks above and below 0. (B) Normal samples from the kidney cancer dataset were used to generate data with no differential variability. Ten thousand CpGs and 100 samples (50 in each group) were randomly selected.
